# Electroacupuncture Inhibits Pain Memory and Related Anxiety-Like Behaviors by Blockading the GABA_B_ Receptor Function in the Midcingulate Cortex

**DOI:** 10.1007/s12035-023-03467-9

**Published:** 2023-07-19

**Authors:** Xiaoyu Li, Yichen Zhu, Haiju Sun, Zui Shen, Jing Sun, Siqi Xiao, Xiaofen He, Boyu Liu, Yifang Wang, Yuxin Hu, Boyi Liu, Yi Liang, Yongliang Jiang, Junying Du, Chi Xu, Jianqiao Fang, Xiaomei Shao

**Affiliations:** The Third Clinical Medical College, Key Laboratory of Acupuncture and Neurology of Zhejiang Province, Zhejiang Chinese Medical University, No.548 Binwen Road, Binjiang District, Hangzhou, 310053 Zhejiang China

**Keywords:** Pain, Memory, Electroacupuncture, Midcingulate cortex, Rat

## Abstract

Pain memory is commonly considered an underlying cause of chronic pain and is also responsible for a range of anxiety. Electroacupuncture (EA) has been shown to ameliorate pain memories and exert anti-anxiety effects. Previous research has indicated that GABAergic neurons and/or GABA receptors (GABARs) in the midcingulate cortex (MCC) have potential associations with chronic pain and anxiety. However, there is no known empirical research that has specifically studied the effects of EA on the GABAergic system in the MCC. Here, we used cross-injection of carrageenan to establish the pain memory rats model. Immunofluorescence were used to detect the excitability of GABAergic neurons within MCC. Von Frey filament, elevated zero maze, and open field tests were used to measure mechanical allodynia and anxiety-like behaviors, combined with chemogenetic and pharmacologic technologies. Finally, this study provides evidence that pain memories contribute to generalized negative emotions and that downregulating the activity of GABAergic neurons within MCC could block pain memories and reverse anxiety emotion. Specifically, GABA_B_R is involved in pain memory and related anxiety-like behaviors. Activation of GABAergic neurons in the MCC did not reverse the effects of EA on pain memories and related anxiety-like behaviors, whereas these effects could be reversed by a GABA_B_R agonist. These findings highlight the functional significance of GABA_B_R in the EA-mediated attenuation of pain memories and related anxiety-like behaviors in rats.

## Introduction

One-fifth of the global population suffers from chronic pain, which has emerged as a significant worldwide health concern [1]. Persistent pain is a major characteristic of clinical pain disorders, and it is difficult to separately assess pain from the co-occurring cognitive and emotional features in chronic pain patients [2]. Researchers have attributed chronic pain to a maladaptive memory mechanism [3]. And chronic pain has also been characterized as the enduring presence of a pain memory trace and/or the incapacity to eliminate a pain memory elicited by an initial injury [4, 5]. In line with this idea, a painful event is strongly influenced by our prior pain experience and repeated pain stimuli. Our prior study found that pain memory induced an aversive response to previously paired pain produced by carrageenan injection [6]. Further documentation reported that affective responses elicited by unfavorable feedback, as opposed to impartial feedback, were linked to self-reported symptoms of anxiety and intrusive ideation [7]. Additionally, repeated stimulation can create implicit memory patterns and abnormal stimulus–response sequences that might contribute to the development of anxiety or hyperalgesia syndrome in patients [8]. However, the critical neurobiological mechanism underlying pain memory–related generalized anxiety-like behaviors is poorly understood.

Clearly, the brain contains specific regions that are responsible for encoding a variety of functions; however, several hub areas display a noticeable convergence of overlapping functions, with the middle cingulate cortex (MCC) being particularly noteworthy. Increasing evidence from animal and clinical investigations has confirmed multiple functions of MCC, including cognitive control [9], pain [10], emotional processing [11], and decision-making [12]. Given the relationship between negative emotion and pain, the contribution of MCC to pain memory–related generalized anxiety-like behaviors should be considered and explored.

GABAergic neurons synthesize the neurotransmitter gamma-aminobutyric acid (GABA), which is the most important inhibitory neurotransmitter in the brain responsible for regulating neuronal excitability. The role of GABAergic neurons is broad and complex [13], and the activation or inhibition of GABAergic neurons in specific brain regions through various GABA receptors may lead to diverse results [14, 15]. Moreover, GABA neurotransmission regulates the excitability of pyramidal neurons and interneurons through feedforward inhibition, feedback inhibition, and other inhibition mechanisms to change the direction of information transmission [16]. In the state of chronic pain, GABAergic neurons undergo synaptic plasticity, and several studies have suggested the involvement of GABAergic neurons in pain symptoms and associated negative emotions [17]. In addition, chemogenetic activation of GABAergic neurons improved anxiety-like behaviors [18, 19]. Few studies have investigated the effects of GABAergic neurons within the MCC on pain memory–related negative emotional responses.

Electroacupuncture (EA) has been widely recognized as a therapy for persistent pain [20, 21] and anxiety [22, 23]. EA, in a series of our previous studies, not only showed analgesic effects, but also disrupted the pain memory retrieval of the rats, by partial inhibition of cAMP/PKA/CREB signaling [24]. However, the detailed mechanism by which EA regulates negative emotions, such as anxiety, that are induced by pain memory is still ambiguous.

Therefore, we executed the present study to confirm that negative emotional responses are induced by pain memory and to explore the possible mechanisms by which GABAergic neurons and GABA receptors (GABARs) in the MCC may be involved in the processing of negative emotions in connection with pain memory.

## Materials and Methods

### Animals

Adult male Sprague–Dawley rats, aged between 7 and 8 weeks and weighing 250–300 g, were supplied by the Laboratory Animal Center of Zhejiang Chinese Medical University, which is the Association for Assessment and Accreditation of Laboratory Animal Care (AAALAC)-accredited. The rats were certified by the AAALAC. The rats were housed under suitable conditions with three rats per cage in a temperature-controlled environment of 23–25 °C and 40–60% humidity under a 12-h light/dark cycle. Rats had unlimited access to food and water. Before commencement of the experiment, the rats were fed an adaptive diet for at least one week. The Animal Ethics Committee of Zhejiang Chinese Medical University approved all experiments.

### Preparation of Adeno-Associated Viruses

Adeno-associated viruses (AAV2/9) were designed to achieve the chemogenetic manipulation strategy: rAAV-VGAT1-mCherry-WPRE-pA (PT-0325) combined with rAAV-VGAT1-hM3D(Gq)-mCherry-WPRE-pA (PT-489) or rAAV-VGAT1-hM4D(Gi)-mCherry-WPRE-pA (PT-488) was designed to excite or inhibit GABAergic neurons of MCC. rAAV-VGAT1-mCherry-WPRE-pA was selected as the control virus. All the viruses were acquired from Wuhan BrainVTA Scientific and Technical Corporation.

### Viral Injections

Rats were anesthetized with isoflurane (output concentration 2%, oxygen flow 500 mL/min). The temperature was maintained and monitored with a thermometer during the operation. A midline incision of the skull was made to fully expose the bregma and lambda areas. According to a standard rat brain atlas (http://labs.gaidi.ca/rat-brain-atlas/), the medial cingulate cortex (− 0.6 mm anterior to Bregma, ± 0.6 mm mediolateral, 1.8 mm dorsoventral) was located for viral injections. The desired viral vectors (450 nL) were injected into the MCC at a rate of 40 nL per 60 s. If the viral infection area exceeded the MCC region, it was not included in the statistics.

### Chemogenetic Manipulation

According to the experimental requirements of each group, the MCC was microinjected with the viral vectors. The control group, model group, and EA group were injected with a control virus vector (rAAV-VGAT1-mCherry-WPRE-pA) without functional elements. The control + hM3D (C + hM3D) group, model + hM3D (M + hM3D) group, and EA + hM3D (EA + hM3D) group were injected with rAAV-VGAT1-hM3D(Gq)-mCherry-WPRE-pA containing hM3D (excitatory) functional elements. The C + hM4D group and M + hM4D group were injected with rAAV-VGAT1-hM4D(Gi)-mCherry-WPRE-pA, which contained hM4D (suppressive) functional elements.

Moreover, chemogenetic manipulation was combined with mechanical pain threshold detection. The rats were exposed to the pain threshold detection room for a duration of 30 min and then injected intraperitoneally (i.p.) with clozapine N-oxide (CNO) solution. The concentration of CNO was 1 mg/mL, and the dose was 2 mg/kg. The time points of injection were 4.5 h and 1–5 days after the first injection. Mechanical pain threshold detection was performed 30 min after CNO i.p. injection.

### Pharmacology

#### Cannulation Surgery

One week prior to the start of the experimental protocol, aseptic surgeries were measured with the rats. Bilateral guide cannulae aimed at the medial aspect of the MCC (from bregma: − 0.6 mm anterior to bregma, ± 0.6 mm mediolateral, 1.8 mm dorsoventral) were stereotaxically implanted in rats under isoflurane (output concentration 2%, oxygen flow 500 ml/min) anesthesia.

#### Intracerebral Microinjection of Drugs

The model rats and EA rats were injected with a vehicle, and the M + AR antagonist group, M + BR antagonist group, and EA + BR agonist group were injected with bicuculline, CGP55845, and baclofen, respectively, at 4.5 h after the first modeling and 1 day, 2 days, 3 days, 4 days, and 5 days after the first injection.

### Model

The model rats were developed by the cross injecting of carrageenan. The specific method was as follows: carrageenan (2%, 100 μl) was first injected into the center of the plantar surface of the left hind limb. Fourteen days after the first injection, the second injection was measured into the right rear plantar bottom of rats after the pain threshold was recovered. Carrageenan powder was dissolved in a 2% solution of normal saline and stored at 4 °C before use. The control group animals were given 100 μl saline.

### Pain Withdrawal Thresholds

To assess paw withdrawal thresholds (PWTs), a dynamic plantar Von Frey hair esthesiometer (Ugo Basile, Comerio, Italy) was used. Before test, rats were placed in a testing chamber and permitted to acclimatize for 30 min. A stainless-steel probe was placed beneath the mesh floor, positioned perpendicular to the center of the plantar surface of the left hind paw. The probe was subjected to an incremental increase of vertical force, from 0 to 50 g at a rate of 2.5 g/s, with careful positioning. Each application of the probe lasted for a period of 5 s or until positive responses of paw lifting or licking were observed. PWTs were recorded at baseline, 4 h, 1 day, 3 days, 5 days, and 13 days after the first carrageenan injection and 4 h, 1 day, and 3 days after the second carrageenan injection.

### Assessment of Anxiety-Like Behaviors

The behavioral tests were conducted in a darkroom, with the temperature maintained at 23–26 °C and the humidity at 40–60%. Prior to experiments, rats were placed in the environment for habituation measurement. Rats’ activities were recorded and analyzed using the ANY-Maze animal behavior video tracking and analysis system. The behavioral tests were commenced 30 min after CNO or 0.9% saline injection.

### Elevated Zero Maze

The Elevated Zero Maze (EZM) test was conducted 1 day after the second injection of carrageenan. The current study employed a maze consisting of two closed arms as well as two open arms to assess behavioral patterns. Following transfer to a low-lit experimental chamber, rats were placed on an open arm for a 30-s adaptation period, upon which the mouse’s activity was monitored for at least 5 min. To prevent olfactory cue bias, the maze apparatus was thoroughly washed with 75% alcohol following each test. The anxiety-like behaviors of the rats were recorded and analyzed using the Any-Maze software. All data recorded within the 5 min were included in the analysis. And the data were exported to SPSS for further statistical analysis.

### Open Field Test

The open field test (OFT) was conducted for each group of rats 16 days after modeling. The opening field was a 1 m × 1 m × 50 cm opaque black cuboid with no lid, and the surface area of the bottom of each box was evenly divided into 16 small cells. The mice were cautiously placed in the center of an open field arena. Each rat was individually transported into a dimly lit testing chamber where they were left undisturbed for 30 s to become acclimated to the environment before activity recordings were made for 5 min. To accurately compare results between light-on and light-off conditions, the rats were placed in the center of the open field for each test and total distance traveled, as well as the percentage of time spent in the center zone was analyzed. The anxiety-like behaviors of the rats were recorded and analyzed using professional software (Any-Maze). All data recorded within the 5 min were included in the analysis. And the data were exported to SPSS for further statistical analysis.

### EA

Without anesthesia, the rats in the EA group, the hM3D + EA group, and the BR agonist + EA group received EA intervention in the acupuncture point (Zusanli) ST36, located below the knee along the bilateral stomach meridian, and the reference electrode (1 cm inferior to ST36) at 5 h, 1 day, 2 days, 3 days, 4 days, and 5 days after establishment of the pain memory model. The method of point positioning is referenced in the “Acupoint Atlas of Experimental Animals.” The EA intervention was performed using acupuncture needles and a HANS Acupuncture Point Nerve Stimulator. The model of Acupuncture Point Nerve Stimulator was LH-202H, made in Huawei Co., Ltd. (Beijing, China). The needles were 0.25 mm in diameter × 13 mm in length. The EA frequency was set at 2/100 Hz, and the intensity was gradually increased from 1 to 2 mA at 0.5 mA increments every 10 min, with a total treatment duration of 30 min. To ensure a relaxing effect, black hoods were placed over each animal’s head during the EA intervention. All subjects appeared completely at ease, and no signs of stress were observed. The other rat groups that did not receive EA administration wore black hoods for 30 min simultaneously.

### Statistical Analysis

For data analysis, mean ± SEM were the standard parameters. Repeated-measure analysis of variance (ANOVA) was employed to determine the mechanical withdrawal thresholds for each group. The least significant difference and Dunnett’s test were used in post hoc analysis, corresponding to equal or unequal variances. One-way ANOVA testing was conducted on the results of open field and elevated O maze experiments to compare different groups. A *P* value < 0.05 was considered statistically significant.

## Results

### Pain Memory Induced by a Second Cross Injection of Carrageenan Yielded Anxiety-Like Behaviors and the Elevation of GABAergic Neurons in the MCC

**Fig. 1:**
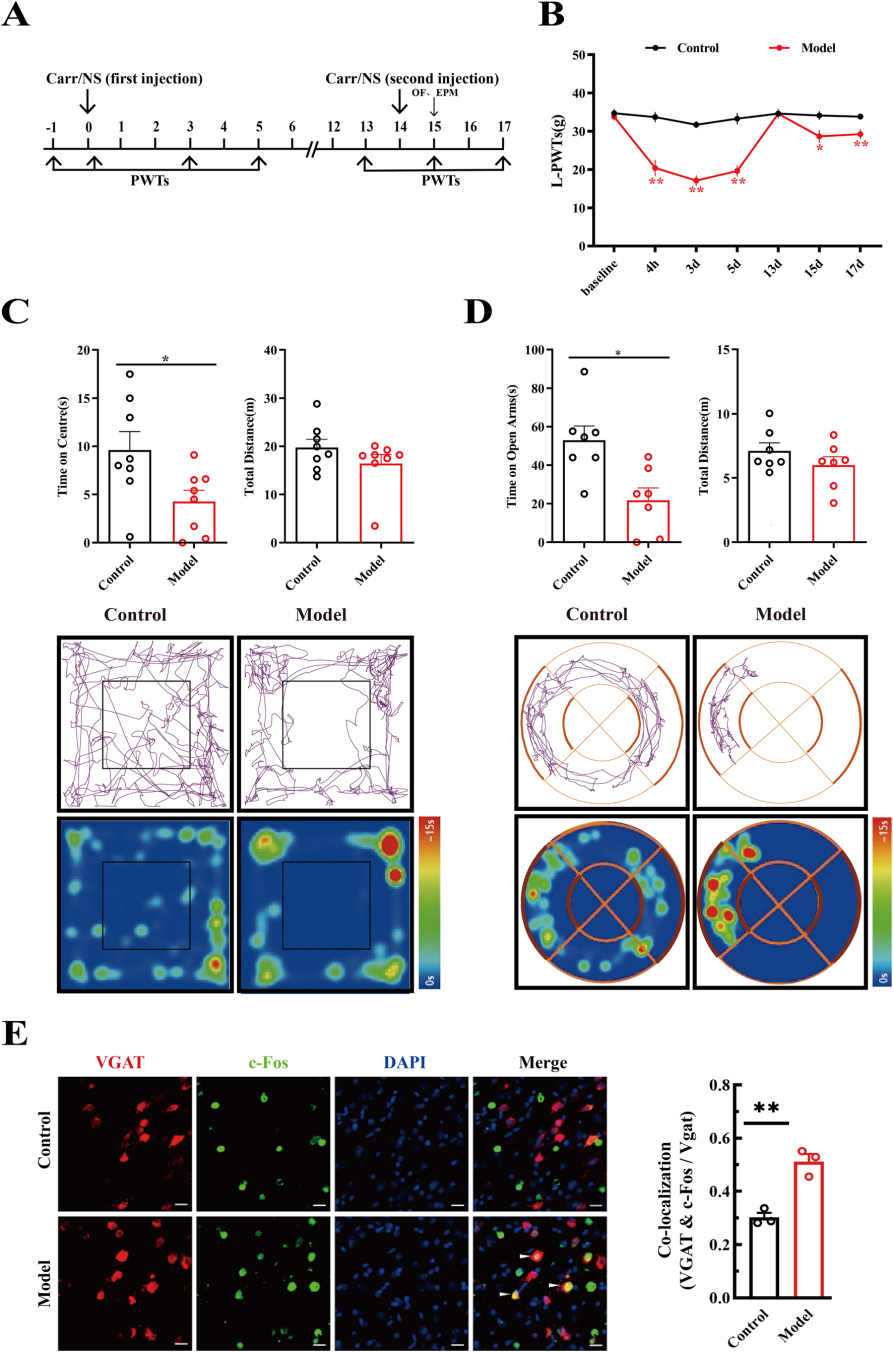
The pain memory model rats showed anxiety-like behaviors and enhanced GABAergic neuron excitability. **A** The figure shows the daily experimental timeline. **B** The comparison between control and model group of the left-paw withdrawal thresholds. * represents statistically significant. *P < 0.05, **P < 0.01 (compared with the control group). **C** The results of open field test (OF). * represents statistically significant. *P < 0.05 (compared with the control group). **D** The results elevated zero maze test (EZM). * represents statistically significant. *P < 0.05 (compared with the control group). **E** Left figure, representative figure of the colocalization of c-Fos (green) with GABAergic (red) neurons. Arrows show colocalization of c-Fos with neurons. Scale bar, 500 μm

The pain memory model was performed using a previously reported procedure with minor modifications [25]. In this part of the experiment, a decreased mechanical pain PWT of the left hind paw after the second cross-injection represented a successfully established pain memory rat model, and the left hind paw was called the pain memory paw. EA treatment was performed at 4 h and days 1–5 after the first injection. Anxiety-like behaviors were examined 15–17 days after the second carrageenan injection by using the OFT and EZM. The detailed time schedule of this part is depicted in Fig. 1A.

A single carrageenan injection is a commonly used method to establish an acute inflammatory pain model [26]. As shown in Fig. 1B, after the first injection, the left hind paw PWTs (L-PWTs) of rats were decreased compared with those of the control group 4 h after the first carrageenan injection and persisted for at least 5 days (*P* < 0.01). Thirteen days after the first carrageenan injection, the L-PWTs of the model group rats were restored to a normal level compared to those of the control group (*P* > 0.05), and the second cross-injection was performed on the right hind paws. Compared with the control group, the PWTs of the left hind paw, which were not subjected to the second carrageenan injection, in model rats lowered again, indicating that the 2nd cross-injection evoked pain memory in rats.

Furthermore, pain experience involves different dimensions, such as negative emotions. We wondered whether pain memory model rats would exhibit anxiety-like behaviors. Thus, we conducted OFT and EZM experiments on the rats. As expected, significant differences between the control and model groups were found in the time spent in the center square in the OFT (Fig. 1C). Additionally, the time spent in the open arm of the EZM was significantly different (Fig. 1D). Total distances moved in both the OFT and EZM experiments showed no significant differences among groups (Fig. 1C, D).

To define the role of GABAergic neurons within the MCC, we next examined the expression of c-Fos and VGAT in the MCC by immunofluorescence techniques. As shown in Fig. 1E, the percentage of c-Fos colocalization with VGAT in the model group was significantly higher than that in the control group (*P* < 0.01). Together, these results showed that GABAergic activity within the MCC might have great relevance to pain memory and generalized anxiety-like behaviors.

### Inhibiting, Rather than Activating, the GABAergic Neurons of the MCC Reversed Pain Memory and Related Anxiety-Like Behavior in Model Rats

**Fig. 2:**
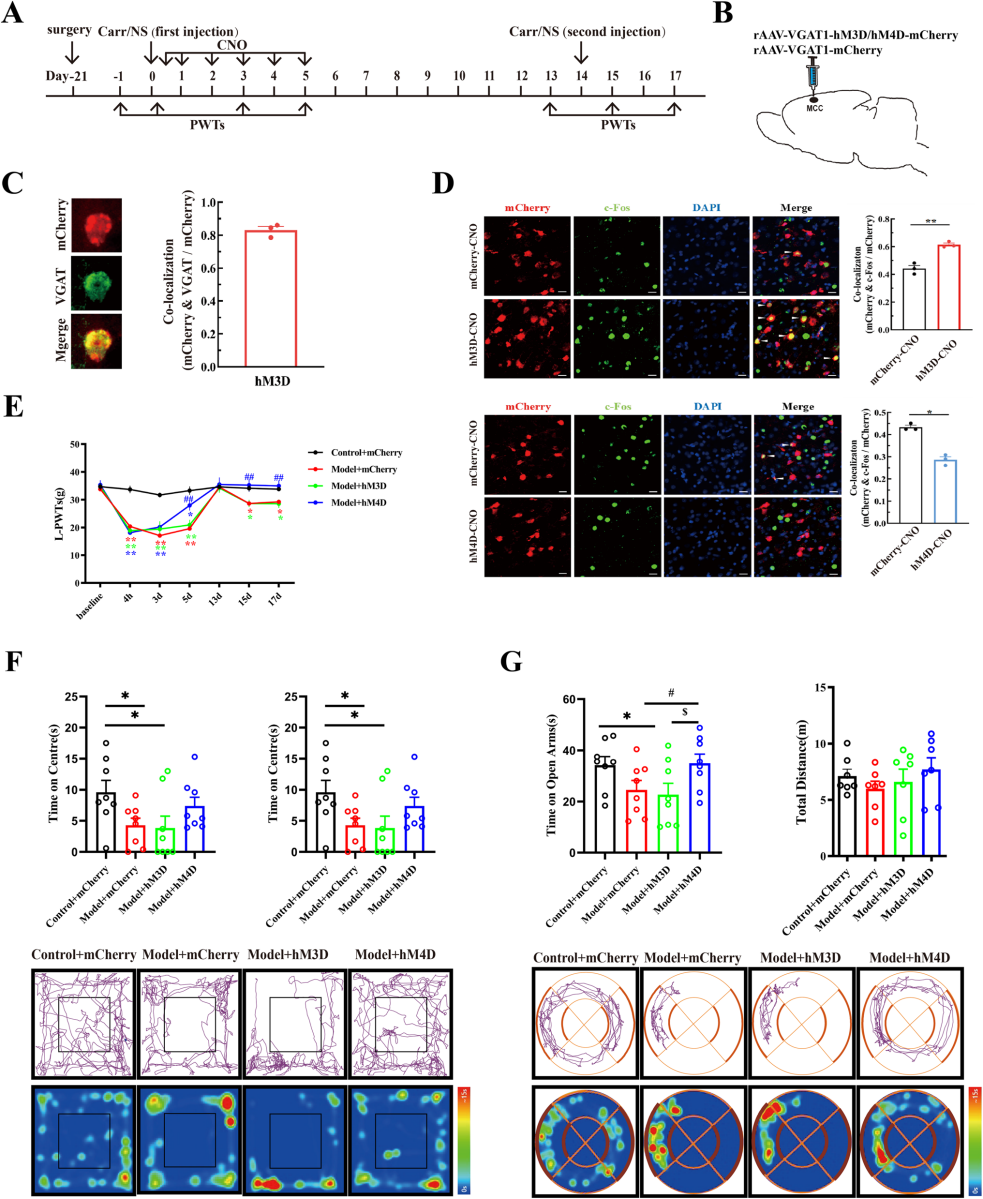
Effects of chemogenetic manipulating MCC GABAergic neurons on pain memory and anxiety-like behavior in rats. **A** The daily experimental timeline. **B** Schematic diagram of the virus injection site of MCC. **C** Green represents the VGAT positive neurons, red represents the virus, co-localization was confirmed by yellow dots. Scale bar, 50 μm. Statistical charts represents the percentage of colocalization (mCherry and VGAT/mCherry). **D** The efficacy tests of hM3Dq and hM4Di viruses. The percentage of colocalization of mCherry (red) and c-Fos (green) were counted. The blue substance was DAPI. * represents statistically significant difference. *P < 0.05, **P < 0.01 (compared with the mCherry + CNO group); scale bar, 500 μm. **E** The comparison of the left-paw withdrawal thresholds of the Control + mCherry group, the Model + mCherry group, the Model + hM3D group, and the Model + hM4D group at different time. Both * and ^#^ represent statistically significant difference. *P < 0.05, **P < 0.01 (compared with the Control + mCherry group); ^##^*P* < 0.01, ^#^*P* < 0.05 (compared with the Model + mCherry group). **F**–**G** The upper part of the figure was divided into the Control + mCherry group, the Model + mCherry group, the Model + hM3D group, and the Model + hM4D group. The detection results of open field experiment and O-maze experiment of rats. The lower part was divided into open field experiment and O-maze experiment of rats in each group, and the heat map representative diagram of motion track was compared with the control group

To further test whether MCC GABAergic neurons are required for pain memory and related anxiety-like behaviors, we chemogenetically activated and inhibited GABAergic neurons in the MCC (Fig. 2A). First, the same amounts of viruses rAAV-VGAT1-hM3D-mCherry, rAAV-VGAT1-hM4D-mCherry, and rAAV-VGAT1-mCherry were injected into the right MCC region (Fig. 2B). Then, immunofluorescence experiments were performed to validate the specificity and efficacy of the viruses. As a result of the viruses expressing mCherry, the colocalization of mCherry with VGAT indicated that viruses specifically infected GABAergic neurons (Fig. 2C). Compared with the mCherry-injected group, the rat groups that were intraperitoneally injected with hM3D increased c-Fos expression in GABAergic neurons, and hM4D injection decreased c-Fos expression (Fig. 2D). We then visualized the effects of inhibiting and activating GABAergic neurons within the MCC on pain memory. As demonstrated in Fig. 2E, the L-PWTs of rats in each group shown significant decreases at 4 h and 3 days after the first injection of carrageenan, compared with those in the Control + mCherry group (*P* < 0.01). Of note, by the fifth day after the first injection, the L-PWTs of rats in the Model + hM4D group exhibited a significant increase, compared with those in the model group (*P* < 0.01), while the rats in the Model + hM3D group were not affected (*P* > 0.05). Once the L-PWTs in each rat recovered to normal levels, a second injection was introduced to the hind paws on the right. In an interesting observation, the L-PWTs of rats in the Model + hM4D group, when compared to those in the Control + mCherry group, indicated no significant decrease (*P* > 0.05), whereas the variation in the L-PWTs of the Model + hM3D group was similar to that in the model group (*P* > 0.05). These results suggest that impeding GABAergic neurons in the MCC has the potential to inhibit the retrieval of pain memory.

As a result of the model rats showing high expression of c-Fos in the MCC, we next conducted OFT and EZM experiments in each group of rats to observe whether the early regulation of MCC GABAergic neurons influences pain memory–related anxiety-like behaviors. During the OFTs, rats in the Model + hM3D group demonstrated a significant decrease in the amount of time spent in the center zone, when compared to the control group (*P* < 0.05). Conversely, the time spent in the center zone for rats in the Model + hM4D group displayed no significant difference relative to that of rats in the control group (*P* < 0.05). Furthermore, the total distance traveled by all rats showed no observable difference between groups (*P* < 0.05), as presented in Fig. 2F. Regarding the EZM tests, rats in the Model + hM4D group spent more time in the open arm of the maze than either the model or Model + hM3D group rats (both *P* < 0.05), while the total distance traveled showed no significant differences among all groups (*P* < 0.05), as shown in Fig. 2G. These results indicated that inhibiting GABAergic neurons in the MCC reversed pain memory and related anxiety-like behavior in model rats, while activating GABAergic neurons had no influence.

### Chemogenetic Activation or Inhibition of GABAergic Neurons in the MCC Had No Effects on Pain Memory and Anxiety-Like Behaviors in Naive Rats

**Fig. 3:**
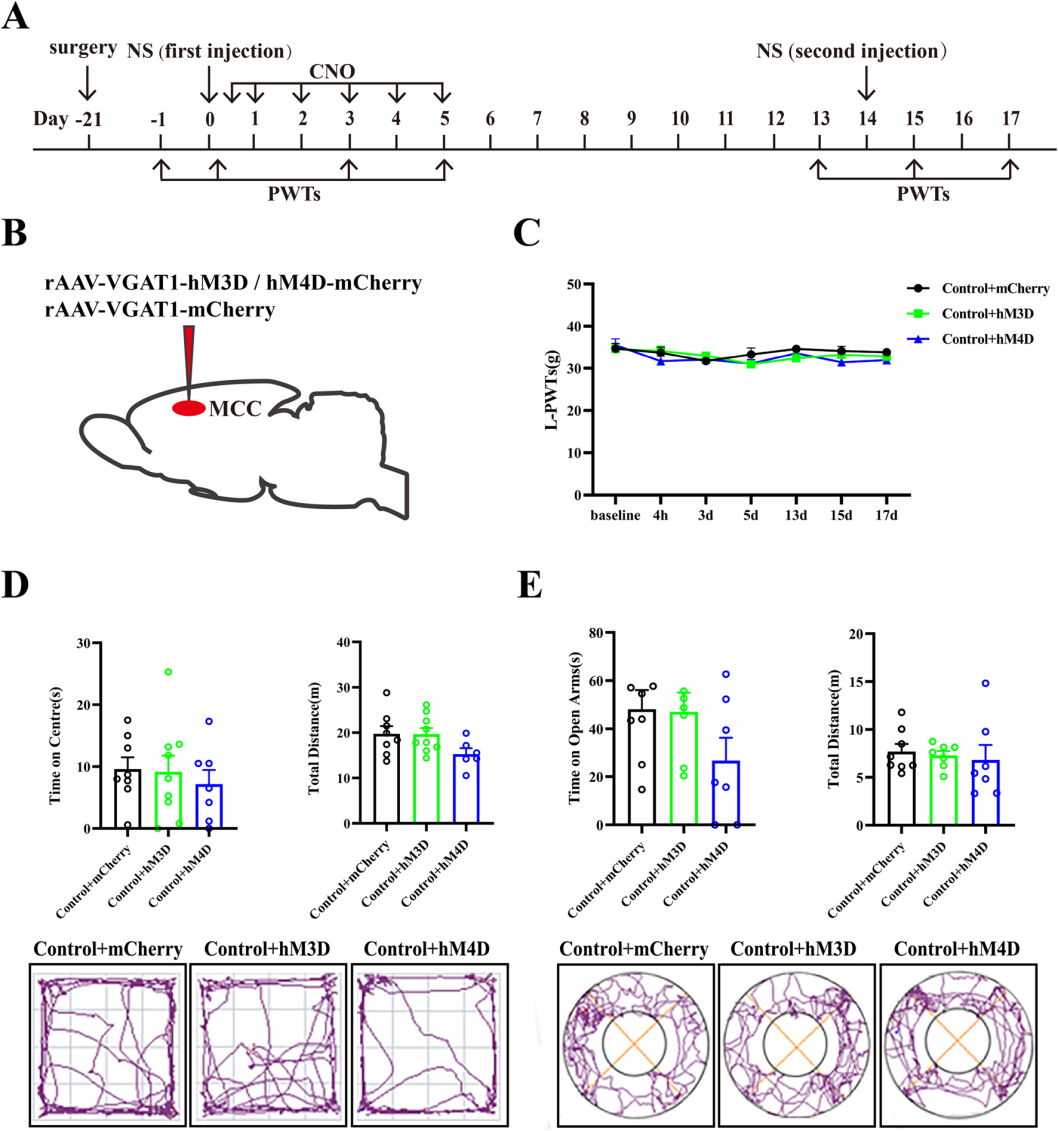
Effects of chemogenetic manipulating MCC GABAergic neurons on pain memory and negative emotions in physiological state. **A** The daily experimental timeline. **B** The plan of the virus injection in the MCC. **C** The L-PWTs of the Control + mCherry group, the Control + hM3D group, and the Control + hM4D group at each time point. **D** The upper part of the figures the test results of the open-field experiment, and the lower parties the representative figure of the movement trajectory of each group of rats in the open-field experiment. **E** The upper part of the figure a representation of the test results of the O maze experiment, and the lower part is a representation of the movement trajectory of each group of rats in the elevated O maze experiment

To further investigate the mechanism of MCC GABAergic neurons in pain memory and related anxiety-like behaviors, we observed whether chemogenetic activation or inhibition of MCC GABAergic neurons in naive mice would influence PWTs, pain memory, and related anxiety-like behaviors. The viruses were microinjected into the MCC region 21 days prior to the first hind paw saline injection. Day 0 saw the administration of 0.1 mL of normal saline to the left hind paws and 0.1 mL of normal saline to the right hind paw on day 14. Thereafter, intraperitoneal administration of CNO was initiated from days 0 to 5, as graphically presented in Fig. 3A and B. Subsequent results showed no significant differences among any group at each given time point (*P* > 0.05), which suggested that chemogenetic activation or inhibition of GABAergic neurons in the MCC have no significant effect on pain memory in naive rats.

Moreover, we also explored the anxiety-like behaviors of each group by OFT and EZM experiments (Fig. 3D, E). In comparison to the Control + mCherry group, rats in the hM3D nor hM4D group exhibited no statistical variants concerning the time spent in the center zone during OFTs (*P* > 0.05) (Fig. 3D). The EZM tests revealed no significant differences in the duration spent in the open arms between all groups (*P* > 0.05) (Fig. 3E). Likewise, there were no significant group differences concerning the total distance traveled (*P* > 0.05). These results implied that chemogenetic activation or inhibition of GABAergic neurons in the MCC did not cause pain memory, nor had any effect on pain memory and anxiety-like behaviors in naive rats.

### Effects of Different GABA Receptors in the MCC on Pain Memory and Anxiety-Like Behaviors

Although the central roles of MCC GABAergic neurons in pain memory and anxiety-like behaviors are clearly defined, it remains unknown whether all GABA receptor types play a critical role. Two types of GABA receptors have been identified: GABA_A_ receptors (GABA_A_Rs) that activate chloride ion channels and GABA_B_ receptors (GABA_B_Rs) that interact with the G-protein-coupled chloride ion channels.

**Fig. 4:**
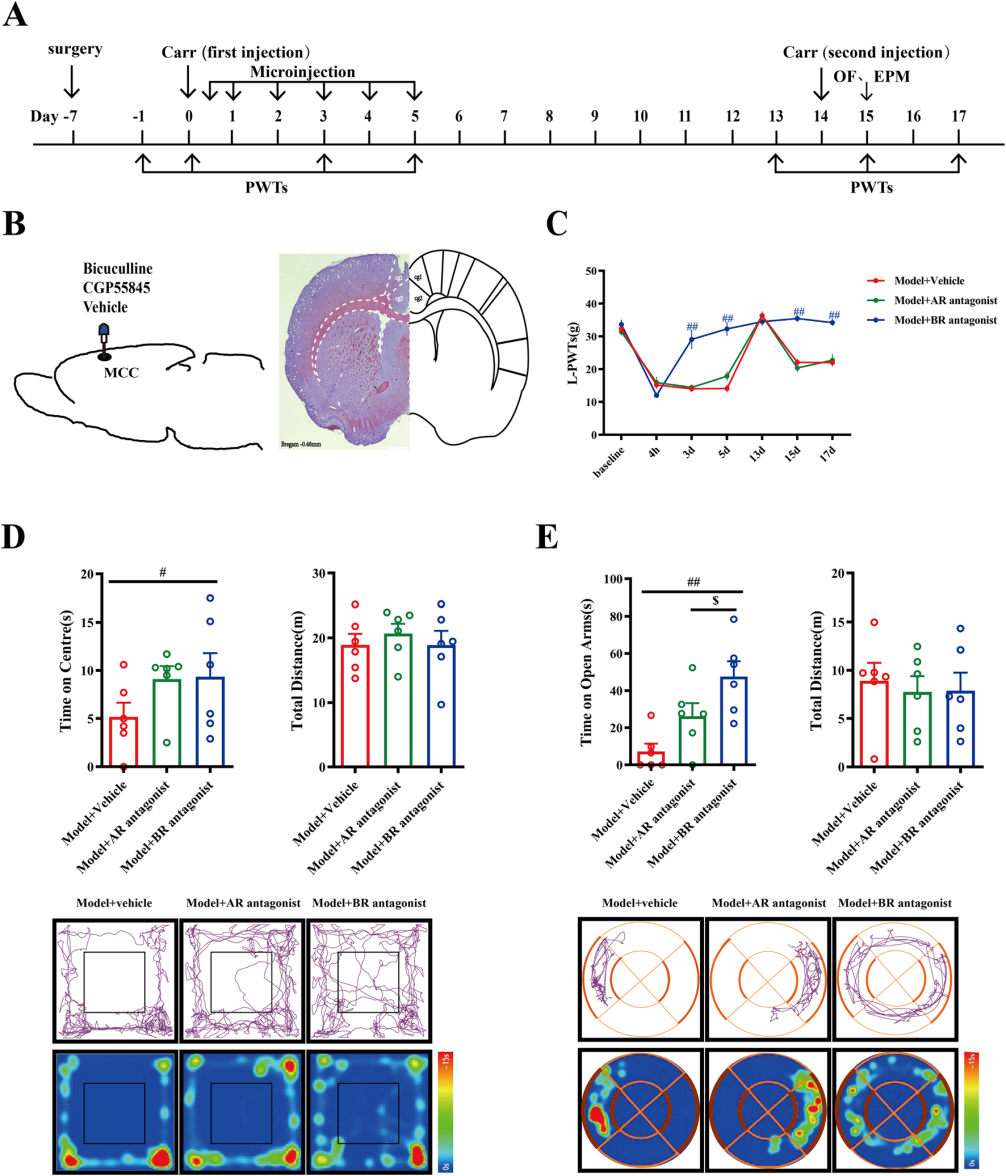
The activation of GABAR in MCC induces pain memory recall and negative emotion. **A** The daily experimental timeline. **B** The plan of the drug injection in the MCC and Nissl staining of MCC catheterization position under type microscope. **C** The comparison of the left-paw withdrawal thresholds of the Model + Vehicle group, the Model + AR antagonist group, and the Model + BR antagonist group at different time. ^#^ represents statistically significant difference. ^##^*P* < 0.01 (compared with the Model + Vehicle group). **D** The upper part of the figures the test results of the open-field experiment, and the lower part is the representative figure of the movement trajectory of each group of rats in the open-field experiment. ^#^ represents statistically significant difference. ^#^*P* < 0.05 (compared with the Model + Vehicle group). **E** The upper part of the figure a representation of the test results of the O maze experiment, and the lower part is a representation of the movement trajectory of each group of rats in the elevated O maze experiment. Both ^#^ and ^$^ represent statistically significant difference. ^##^*P* < 0.01 (compared with the Model + Vehicle group), ^$^*P* < 0.05 (compared with the Model + AR antagonist group)

To explore this issue, 500 nL of GABA_A_R or GABA_B_R antagonists (bicuculline or CGP55845) was microinjected into the right MCC region of the model rats from the fourth hour to the fifth day after the first carrageenan injection (Fig. 4A, B). As shown in Fig. 4C, the pattern of L-PWTs and anxiety-like behavior in the Model + Vehicle group rats was consistent with that in the previous experiments above. The mechanical pain L-PWTs and anxiety-like behavior of GABA_A_R antagonist-treated rats were almost equal to those of model rats at this stage (*P* > 0.05). However, the mechanical pain L-PWTs in the GABA_B_R antagonist group were significantly upregulated from the fourth hour to fifth day after treatment compared with those in the model group. Moreover, Model + GABA_B_R antagonist group rats exhibited pain memory retrieval dysfunction (Fig. 4C). In terms of anxiety-like behaviors, GABA_B_R antagonists improved the time spent in the central zone and open arm of the pain memory rats compared with those of the model rats (*P* < 0.05) (Fig. 4D, E).

Taken together, these results indicated that GABA_B_R inhibition plays a critical role in pain memory and anxiety-like behaviors and that inhibition of GABA_B_R activity fails to impair pain memory and anxiety-like behaviors, while GABA_A_R is not involved in the mechanism.

### The Effects of EA Could Be Blocked by GABAB Receptor Agonists but not by GABAergic Neuron Activation in the MCC

**Fig. 5:**
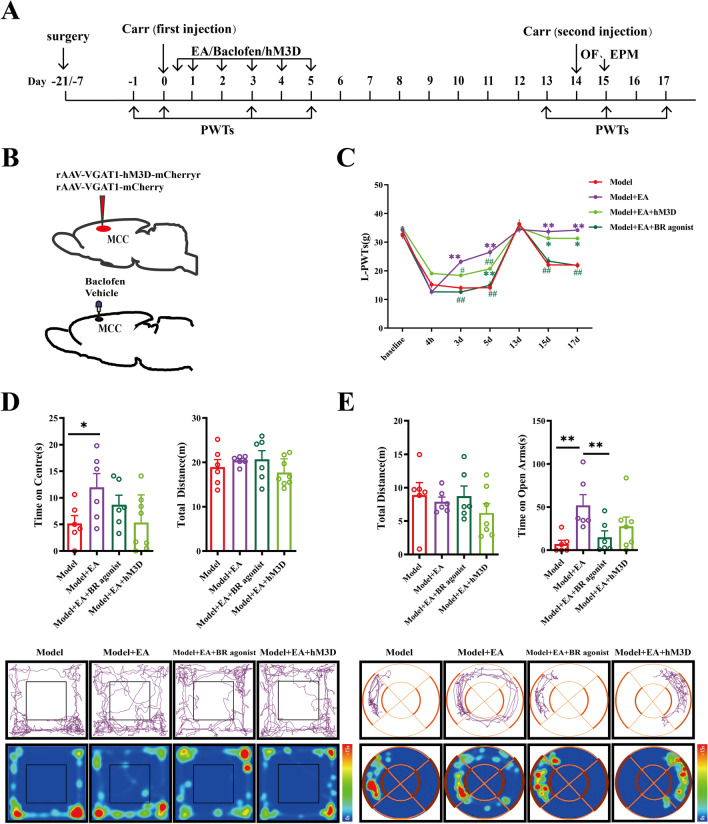
Electroacupuncture partially regulates pain memory through MCC GABAergic neurons and GABA_B_R. **A** The daily experimental timeline. **B** The plan of the drug and virus injection in the MCC. **C** The comparison of the left-paw withdrawal thresholds of the Model group, the Model + EA group, the Model + hM3D + EA group, and the Model + GABA_B_R agonist + EA group at different time. Both * and ^#^ represent statistically significant difference. *P < 0.05, **P < 0.01 (compared with the Model group); ^##^*P* < 0.01, ^#^*P* < 0.05 (compared with the Model + EA group). **D** The upper part of the figures the test results of the open-field experiment, and the lower part is the representative figure of the movement trajectory of each group of rats in the open-field experiment, *P < 0.05. **E** The upper part of the figure a representation of the test results of the O maze experiment, and the lower part is a representation of the movement trajectory of each group of rats in the elevated O maze experiment, **P < 0.01

Our previous studies have shown the blocking effects of EA in blocking pain memory and ameliorating anxiety-like behaviors [24, 27]. However, the mechanisms of EA are still not clear. We further investigated whether EA could regulate the activity of GABAergic neurons and GABA_B_R function. Thus, we microinjected the AAV-VGAT-hM3D-mCherry virus or a GABA_B_R agonist, baclofen, into the MCC of model rats (Fig. 5A, B). As in the previous experimental results, EA administration showed analgesic effects after the first injection and prevented the retrieval of pain memories. Moreover, both the activation of GABAergic neurons and GABA_B_R agonists significantly blocked the analgesic effects of EA after the first injection. Notably, GABA_B_R agonists also blocked the effects of EA in preventing the retrieval of pain memories, while GABAergic neuron activation had no such effects (Fig. 5C).

Finally, we next tested whether EA administration exerted anti-anxiety effects by regulating the activity of GABAergic neurons or GABA_B_Rs. For this purpose, we also performed OFT and EZM experiments on rats with the combined treatment of EA and AAV-VGAT-hM3D-mCherry virus or baclofen (Fig. 5D, E). Consistently, EA administration increased the time the rats spent in the center zones (*P* < 0.05) and the open arms (*P* < 0.01). Notably, baclofen decreased the time spent in the open arms by EA-treated rats (*P* < 0.05). In contrast, hM3D intervention had no effects on EA treatment, neither on the time in the center zones (*P* > 0.05) nor on the open arms (*P* > 0.05).

Thus, the presented data supported that within the MCC, GABA_B_R inhibition, not GABAergic neuron activation, is the key mechanism by which EA blocks pain memory and related anxiety-like behaviors.

## Discussion

Previous studies have demonstrated that GABA neurotransmitters and their associated receptors play a crucial role in pain sensitivity, along with the associated negative emotions [28, 29]. Moreover, our previous study discovered a close association between pain relief and anxiety-like behaviors induced by EA, particularly with respect to PV interneurons, which are a distinct subclass of GABAergic neurons situated in the anterior cingulate cortex [13]. However, the mechanisms of different GAB_A_Rs in the MCC in response to EA intervention are still unknown. The present study provides evidence that pain memory contributes to generalized negative emotions, and downregulating the activation of GABAergic neurons in the MCC showed the effects of blocking pain memory and alleviating anxiety. Moreover, GABA_B_R within the MCC is involved in pain memory and related anxiety-like behaviors. The effects of EA on pain memory and related anxiety-like behaviors could be reversed by GABA_B_R agonists, not by activation of GABAergic neurons, in the MCC. Therefore, it is conceivable to propose a novel mechanism-based therapeutic strategy that targets pain-related emotional disorders.

There is convincing evidence that shows the association between the MCC, chronic pain, and negative emotion. A previous study using magnetic resonance imaging indicated that the main region of activation associated with pain management is the MCC band area [30], and the presence of anxiety mediated the nodal efficiency of MCC [31]. Further research proved that the glutamatergic neurons of the MCC were inhibited by pain stimulation in rodents [10]. However, there is still a need for further investigation into the involvement of GABA neurons in chronic pain and anxiety. In this study, we conducted experiments using a well-established animal model to explore pain memory, which is a key factor in contributing to pain development or maintenance. By administering a second cross-injection of carrageenan, we successfully induced anxiety-like behaviors in our animal subjects. Through an immunofluorescence assay, we confirmed that the excitation of GABAergic neurons within MCC was enhanced in pain-memory-model rats with anxiety-like behaviors. Although chemogenetic activation or inhibition of GABAergic neurons in the MCC of normal rats did not show any intervention effects, suppression of GABAergic neuronal activity in the MCC of the model rats not only blocked pain memory but also attenuated the related anxiety-like behaviors, suggesting that the activity of GABAergic neurons may be the crucial mechanism of pain memory and related anxiety.

GABAergic neurons secrete the inhibitory transmitter GABA, which is activated via GABARs. The central nervous system has two primary types of GABA receptors—ionotropic GABA_A_Rs and metabotropic GABA_B_Rs [32]. Drugs impact GABAergic system function, and are commonly used to treat anxiety symptoms, targeting both GABA_A_Rs and GABA_B_Rs [33]. However, the mechanisms by which GABARs in the MCC affect pain memory–related anxiety remain poorly characterized. Thus, we microinjected GABA_A_R and GABA_B_R antagonists within the MCC region. The administration of CGP55845, a GABA_B_R antagonist, was able to prevent pain memory and anxiety, which were not observed in rats injected with bicuculline, a GABA_A_R antagonist. These results suggested that MCC GABA_B_Rs play a crucial role in pain memory and related anxiety.

Numerous clinical and preclinical studies have pointed out the positive effects of EA in the management of pain memory and anxiety. Our current study builds upon previous research that demonstrates the efficacy of EA treatment in blocking pain memory [34]. Additionally, we provide evidence to support the notion that EA can also effectively release the anxiety associated with pain memory. Our previous study also suggested that the administration of EA effectively suppressed the retrieval of aversive memory and alleviated aversive behaviors related to pain memory by reducing the levels of theta power in the rostral ACC [6]. Regarding anxiety, GABAergic neurons are considered to be involved in the process of EA intervention. Studies have suggested that EA treatment ameliorates pain-related anxiety by activating PV neurons within ACC [13]. However, the mechanisms of GABAergic neurons within the MCC remain primarily undefined. Therefore, we then observed an inversion in the anti-anxiety effects of EA treatment after activating GABAergic neurons by using chemogenetic technology, while the analgesic effect of EA was unaffected. Considering that GABA contributed to pain memory–related anxiety through GABA_B_R within the MCC, we then wondered whether EA stimulation acts through GABA_B_R within the MCC. Baclofen, as a GABA_B_R agonist, is a highly effective muscle relaxant and is often used clinically to relieve spasms by intrathecal injection [35]. And oral baclofen was proved to be effectiveness in reducing harmful drinking [36]. However, whether baclofen can affect the pharmacological action of GABA_B_Rs in MCC was still lacking further investigation. Considering the above results, we speculated that baclofen would reverse the effect of EA. Thus, we microinjected baclofen into the MCC, combined with EA intervention. Thus, the GABA_B_R agonist baclofen was microinjected into the MCC. The results showed that the microinjection of baclofen reversed both the analgesic and anti-anxiety effects of EA treatment. As we envisaged, baclofen significantly blocked the analgesic effects and anti-pain memory effects of EA. It also decreased the time spent in the open arms by EA-treated rats. Admittedly, it was a limitation of this study that we did not microinject baclofen into MCC alone, to further explore the effects of MCC GABA_B_Rs on pain and anxiety behavior in rats. But our results were sufficient to prove that GABA_B_R played a critical role on pain memory and anxiety-like behaviors, and GABA_B_R inhibition played the key mechanism of EA blocks pain memory and related anxiety-like behaviors.

Overall, these results suggested that the analgesic and anti-anxiety effects of EA on pain memory model rats may be mediated by activation of GABA_B_Rs within the MCC.

## Conclusion

The present study provides evidence that pain memory contributes to generalized negative emotions and that downregulating the activation of GABAergic neurons within MCC could reverse pain memory and related anxiety-like behaviors. Moreover, the GABA_B_R is involved in pain memory and related anxiety-like behaviors. The effect of EA on pain memory and related anxiety-like behaviors could be reversed by a GABA_B_R agonist, not by activation of GABAergic neurons, in the MCC.

## Data Availability

The original contributions presented in the study are included in the article; further inquiries can be directed to the corresponding author.

## Abbreviations

*MCC*: Middle cingulate cortex
*GABA*: Gamma-aminobutyric acid
*GABARs*: Gamma-aminobutyric acid receptors
*EA*: Electroacupuncture
*SD*: Sprague-Dawley
*CNO*: Clozapine N-oxide
*PWTs*: Paw withdrawal thresholds
*EZM*: Elevated zero maze
*OFT*: Open field test
*MRI*: Magnetic resonance imaging
